# [μ-10,21-Dimethyl-3,6,14,17-tetra­aza­tricyclo­[17.3.1.1^8,12^]tetra­cosa-1(23),8(24),9,11,19,21-hexa­ene-23,24-diolato-κ^8^ *N* ^3^,*N* ^6^,*O* ^23^,*O* ^24^:*N* ^14^,*N* ^17^,*O* ^23^,*O* ^24^]bis­[(nitrato-κ^2^ *O*,*O*′)nickel(II)]

**DOI:** 10.1107/S1600536809010174

**Published:** 2009-04-18

**Authors:** Quan-Jun Li, Jian-Fang Ma, Jie Liu, Ting-Ting Han

**Affiliations:** a Department of Chemistry, Northeast Normal University, Changchun 130024, People’s Republic of China

## Abstract

In the title centrosymmetric dinuclear nickel complex, [Ni_2_(C_22_H_30_N_4_O_2_)(NO_3_)_2_], each of the two Ni^II^ atoms has a distorted octa­hedral geometry, defined by two N atoms and two O atoms from the macrocyclic ligand and two O atoms from a chelating nitrate anion. The two Ni atoms are bridged by two phenolate O atoms, forming a four-membered Ni_2_O_2_ ring.

## Related literature

For general background, see: Caldwell & Crumbliss (1998 ▶); Rosa *et al.* (1998 ▶). For related structures, see: Aromi *et al.* (2002 ▶). For the ligand synthesis, see: Mandal & Nag (1986 ▶).
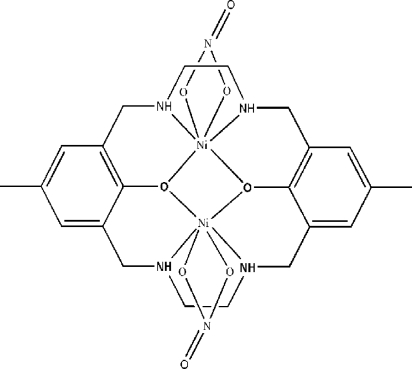


## Experimental

### 

#### Crystal data

[Ni_2_(C_22_H_30_N_4_O_2_)(NO_3_)_2_]
*M* *_r_* = 623.90
Trigonal, 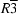

*a* = 25.020 (5) Å
*c* = 10.616 (5) Å
*V* = 5755 (3) Å^3^
*Z* = 9
Mo *K*α radiation
μ = 1.53 mm^−1^
*T* = 293 K
0.40 × 0.30 × 0.25 mm

#### Data collection

Bruker APEX CCD diffractometer
Absorption correction: multi-scan (*SADABS*; Sheldrick, 1996 ▶) *T*_min_ = 0.495, *T*_max_ = 0.609 (expected range = 0.554–0.682)
9432 measured reflections
2213 independent reflections
1745 reflections with *I* > 2σ(*I*)
*R*_int_ = 0.107

#### Refinement

*R*[*F* ^2^ > 2σ(*F* ^2^)] = 0.043
*wR*(*F* ^2^) = 0.117
*S* = 1.03
2213 reflections
178 parameters
1 restraint
H atoms treated by a mixture of independent and constrained refinement
Δρ_max_ = 1.06 e Å^−3^
Δρ_min_ = −0.35 e Å^−3^

### 

Data collection: *SMART* (Bruker, 2007 ▶); cell refinement: *SAINT* (Bruker, 2007 ▶); data reduction: *SAINT*; program(s) used to solve structure: *SHELXS97* (Sheldrick, 2008 ▶); program(s) used to refine structure: *SHELXL97* (Sheldrick, 2008 ▶); molecular graphics: *SHELXTL* (Sheldrick, 2008 ▶); software used to prepare material for publication: *SHELXL97*.

## Supplementary Material

Crystal structure: contains datablocks global, I. DOI: 10.1107/S1600536809010174/hy2188sup1.cif

Structure factors: contains datablocks I. DOI: 10.1107/S1600536809010174/hy2188Isup2.hkl

Additional supplementary materials:  crystallographic information; 3D view; checkCIF report

## Figures and Tables

**Table 1:** Selected bond lengths (Å)

Ni1—O1	2.000 (2)
Ni1—O1^i^	2.006 (2)
Ni1—N2	2.038 (3)
Ni1—N1	2.054 (3)
Ni1—O3	2.134 (3)
Ni1—O2	2.183 (3)

Symmetry code: (i) 
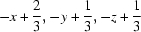
.

**Fig. 1.**
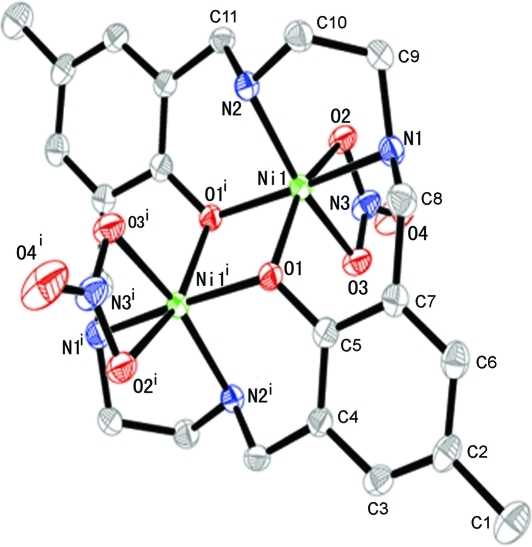
Molecular structure of the title compound. Displacement ellipsoids are draw at the 30% probability level. H atoms have been omitted for clarity. [Symmetry code: (i) -x + 2/3, -y + 1/3, -z + 1/3.]

## supplementary crystallographic information

### Comment

Crown ether compounds have attracted much interest as a result of their significance in biological transport systems (Caldwell & Crumbliss, 1998). In addition, crown ether compounds are found to have functions in selective molecular recognition (Rosa *et al.*, 1998). To further widen the scope of applications of crown ether, there is a need to prepare new series of crown ether compounds. In this work, a new dinuclear nickel(II) compound has been synthesized and its struture is reported here.

As shown in Fig. 1, the title compound is a centrosymmetric dinuclear nickel complex. The coordination environment around each Ni^II^ atom is distorted octahedral, with one N atom and one O atom from the macrocyclic ligand and two O atoms from the nitrate anion occupying the equatorial plane, and the other N atom and O atom from the ligand occupying the axial positions. In the complex molecule, two Ni atoms are bridged by two phenolate O atoms, generating a four-membered Ni_2_O_2_ ring, with a Ni···Ni distance of 2.9737 (10) Å. The Ni—O and Ni—N distances are normal (Aromi *et al.*, 2002).

### Experimental

The macrocyclic ligand, C_22_H_32_N_4_O_2_ (H_2_*L*), was prepared by the reported procedure (Mandal & Nag, 1986). A mixture of H_2_*L* (0.10 g, 0.26 mmol) and Ni(NO_3_)_2_.6H_2_O (0.15 g, 0.52 mmol) in methanol (20 ml) was stirred for 10 min. The resulting solution was filtered. Green single crystals were obtained by slow evaporation of the filtrate at room temperature (yield 56%).

### Refinement

H atoms bound to C atoms were positioned geometrically and refined using a riding model, with C—H = 0.93 (aromatic), 0.97 (CH_2_) and 0.96 (CH_3_) Å and with *U*_iso_ = 1.2(1.5 for methyl)*U*_eq_(C). The imino H atoms were located in a difference Fourier map and refined with *U*_iso_(H) = 0.128 Å^2^. The highest residual electron density was found 1.03Å from Ni1 and the deepest hole 0.76 Å from Ni1.

### Crystal data

**Table:**

[Ni_2_(C_22_H_30_N_4_O_2_)(NO_3_)_2_]	*D*_x_ = 1.620 Mg m^−^^3^
*M**_r_* = 623.90	Mo *K*α radiation, λ = 0.71069 Å
Trigonal, *R*3	Cell parameters from 3000 reflections
Hall symbol: -R 3	θ = 2.4–28.4°
*a* = 25.020 (5) Å	µ = 1.53 mm^−^^1^
*c* = 10.616 (5) Å	*T* = 293 K
*V* = 5755 (3) Å^3^	Block, green
*Z* = 9	0.40 × 0.30 × 0.25 mm
*F*(000) = 2916	

### Data collection

**Table:**

Bruker APEX CCD diffractometer	2213 independent reflections
Radiation source: fine-focus sealed tube	1745 reflections with *I* > 2σ(*I*)
graphite	*R*_int_ = 0.107
φ and ω scans	θ_max_ = 24.9°, θ_min_ = 1.6°
Absorption correction: multi-scan (*SADABS*; Sheldrick, 1996)	*h* = −15→29
*T*_min_ = 0.495, *T*_max_ = 0.609	*k* = −29→23
9432 measured reflections	*l* = −12→12

### Refinement

**Table:**

Refinement on *F*^2^	Primary atom site location: structure-invariant direct methods
Least-squares matrix: full	Secondary atom site location: difference Fourier map
*R*[*F*^2^ > 2σ(*F*^2^)] = 0.043	Hydrogen site location: inferred from neighbouring sites
*wR*(*F*^2^) = 0.117	H atoms treated by a mixture of independent and constrained refinement
*S* = 1.03	*w* = 1/[σ^2^(*F*_o_^2^) + (0.066*P*)^2^] where *P* = (*F*_o_^2^ + 2*F*_c_^2^)/3
2213 reflections	(Δ/σ)_max_ = 0.007
178 parameters	Δρ_max_ = 1.06 e Å^−^^3^
1 restraint	Δρ_min_ = −0.35 e Å^−^^3^

### Fractional atomic coordinates and isotropic or equivalent isotropic displacement parameters (Å^2^)

**Table:**

	*x*	*y*	*z*	*U*_iso_*/*U*_eq_	
Ni1	0.355561 (19)	0.13534 (2)	0.25357 (4)	0.03150 (19)	
C1	0.04882 (19)	−0.0620 (2)	0.3314 (4)	0.0658 (13)	
H1A	0.0488	−0.0770	0.4147	0.099*	
H1B	0.0372	−0.0948	0.2717	0.099*	
H1C	0.0199	−0.0475	0.3276	0.099*	
C2	0.11262 (17)	−0.00973 (17)	0.3005 (3)	0.0431 (9)	
C3	0.12561 (16)	0.01995 (17)	0.1862 (3)	0.0410 (9)	
H3	0.0947	0.0056	0.1255	0.049*	
C4	0.18315 (15)	0.07079 (16)	0.1570 (3)	0.0353 (8)	
C5	0.22847 (15)	0.09460 (15)	0.2510 (3)	0.0347 (8)	
C6	0.16018 (17)	0.01062 (17)	0.3868 (3)	0.0446 (9)	
H6	0.1536	−0.0111	0.4617	0.054*	
C7	0.21738 (16)	0.06230 (16)	0.3656 (3)	0.0374 (8)	
C8	0.26663 (17)	0.08766 (18)	0.4652 (3)	0.0443 (9)	
H8A	0.2746	0.1284	0.4892	0.053*	
H8B	0.2515	0.0614	0.5391	0.053*	
C9	0.37778 (18)	0.13034 (17)	0.5104 (3)	0.0430 (9)	
H9A	0.4112	0.1221	0.4949	0.052*	
H9B	0.3646	0.1196	0.5972	0.052*	
C10	0.40046 (18)	0.19842 (17)	0.4892 (3)	0.0421 (9)	
H10A	0.3704	0.2085	0.5224	0.050*	
H10B	0.4390	0.2230	0.5342	0.050*	
C11	0.47350 (15)	0.23666 (16)	0.3077 (3)	0.0383 (8)	
H11A	0.4824	0.2031	0.3091	0.046*	
H11B	0.5022	0.2685	0.3644	0.046*	
N1	0.32528 (14)	0.09201 (14)	0.4244 (3)	0.0377 (7)	
N2	0.41009 (14)	0.21377 (13)	0.3532 (2)	0.0355 (7)	
N3	0.37310 (14)	0.05216 (15)	0.1596 (3)	0.0479 (8)	
O1	0.28230 (10)	0.14687 (10)	0.23716 (19)	0.0342 (5)	
O2	0.41509 (12)	0.09599 (12)	0.2220 (2)	0.0455 (6)	
O3	0.32302 (12)	0.05220 (12)	0.1480 (2)	0.0459 (6)	
O4	0.38022 (15)	0.01126 (15)	0.1141 (4)	0.0853 (11)	
HN1	0.320 (3)	0.055 (3)	0.423 (6)	0.128*	
HN2	0.401 (3)	0.242 (2)	0.336 (6)	0.128*	

### Atomic displacement parameters (Å^2^)

**Table:**

	*U*^11^	*U*^22^	*U*^33^	*U*^12^	*U*^13^	*U*^23^
Ni1	0.0299 (3)	0.0299 (3)	0.0353 (3)	0.0154 (2)	−0.00202 (16)	0.00065 (16)
C1	0.041 (3)	0.060 (3)	0.081 (3)	0.014 (2)	0.014 (2)	0.004 (2)
C2	0.037 (2)	0.035 (2)	0.052 (2)	0.0137 (17)	0.0119 (16)	−0.0027 (16)
C3	0.033 (2)	0.038 (2)	0.055 (2)	0.0200 (18)	−0.0023 (16)	−0.0069 (16)
C4	0.0299 (19)	0.0322 (19)	0.0451 (18)	0.0165 (16)	0.0011 (14)	−0.0002 (14)
C5	0.0322 (19)	0.033 (2)	0.0424 (18)	0.0193 (17)	0.0038 (14)	−0.0001 (14)
C6	0.044 (2)	0.042 (2)	0.049 (2)	0.022 (2)	0.0114 (17)	0.0067 (16)
C7	0.037 (2)	0.037 (2)	0.0402 (18)	0.0193 (17)	0.0053 (15)	0.0017 (14)
C8	0.043 (2)	0.051 (2)	0.0380 (18)	0.024 (2)	0.0040 (15)	0.0054 (16)
C9	0.047 (2)	0.045 (2)	0.0366 (18)	0.0226 (19)	−0.0053 (15)	0.0029 (15)
C10	0.048 (2)	0.043 (2)	0.0359 (18)	0.0227 (19)	−0.0046 (15)	−0.0049 (15)
C11	0.033 (2)	0.034 (2)	0.049 (2)	0.0181 (17)	−0.0105 (15)	−0.0059 (15)
N1	0.0404 (18)	0.0370 (18)	0.0381 (14)	0.0211 (16)	−0.0010 (12)	0.0024 (13)
N2	0.0366 (17)	0.0327 (17)	0.0383 (15)	0.0182 (15)	−0.0035 (12)	−0.0001 (12)
N3	0.0349 (19)	0.040 (2)	0.067 (2)	0.0180 (16)	−0.0012 (15)	−0.0111 (16)
O1	0.0289 (13)	0.0311 (13)	0.0408 (12)	0.0137 (11)	−0.0007 (10)	0.0025 (10)
O2	0.0341 (14)	0.0377 (15)	0.0634 (16)	0.0171 (13)	−0.0070 (12)	−0.0076 (12)
O3	0.0329 (15)	0.0434 (16)	0.0570 (15)	0.0159 (13)	−0.0059 (11)	−0.0075 (11)
O4	0.058 (2)	0.062 (2)	0.139 (3)	0.0324 (18)	−0.0003 (19)	−0.048 (2)

### Geometric parameters (Å, °)

**Table:**

Ni1—O1	2.000 (2)	C7—C8	1.502 (5)
Ni1—O1^i^	2.006 (2)	C8—N1	1.481 (5)
Ni1—N2	2.038 (3)	C8—H8A	0.9700
Ni1—N1	2.054 (3)	C8—H8B	0.9700
Ni1—O3	2.134 (3)	C9—N1	1.489 (4)
Ni1—O2	2.183 (3)	C9—C10	1.519 (5)
Ni1—Ni1^i^	2.9737 (10)	C9—H9A	0.9700
C1—C2	1.510 (5)	C9—H9B	0.9700
C1—H1A	0.9600	C10—N2	1.483 (4)
C1—H1B	0.9600	C10—H10A	0.9700
C1—H1C	0.9600	C10—H10B	0.9700
C2—C3	1.374 (5)	C11—N2	1.473 (4)
C2—C6	1.382 (5)	C11—C4^i^	1.505 (5)
C3—C4	1.398 (5)	C11—H11A	0.9700
C3—H3	0.9300	C11—H11B	0.9700
C4—C5	1.400 (5)	N1—HN1	0.87 (6)
C4—C11^i^	1.505 (5)	N2—HN2	0.86 (6)
C5—O1	1.336 (4)	N3—O4	1.223 (4)
C5—C7	1.408 (5)	N3—O3	1.260 (4)
C6—C7	1.386 (5)	N3—O2	1.262 (4)
C6—H6	0.9300		
			
O1—Ni1—O1^i^	84.17 (9)	N1—C8—C7	113.5 (3)
O1—Ni1—N2	97.29 (10)	N1—C8—H8A	108.9
O1^i^—Ni1—N2	87.95 (10)	C7—C8—H8A	108.9
O1—Ni1—N1	91.74 (10)	N1—C8—H8B	108.9
O1^i^—Ni1—N1	172.81 (10)	C7—C8—H8B	108.9
N2—Ni1—N1	86.72 (12)	H8A—C8—H8B	107.7
O1—Ni1—O3	99.46 (9)	N1—C9—C10	110.3 (3)
O1^i^—Ni1—O3	91.39 (10)	N1—C9—H9A	109.6
N2—Ni1—O3	163.09 (11)	C10—C9—H9A	109.6
N1—Ni1—O3	95.11 (11)	N1—C9—H9B	109.6
O1—Ni1—O2	158.83 (9)	C10—C9—H9B	109.6
O1^i^—Ni1—O2	92.93 (9)	H9A—C9—H9B	108.1
N2—Ni1—O2	103.57 (11)	N2—C10—C9	110.8 (3)
N1—Ni1—O2	92.99 (11)	N2—C10—H10A	109.5
O3—Ni1—O2	59.57 (10)	C9—C10—H10A	109.5
O1—Ni1—Ni1^i^	42.16 (6)	N2—C10—H10B	109.5
O1^i^—Ni1—Ni1^i^	42.01 (6)	C9—C10—H10B	109.5
N2—Ni1—Ni1^i^	93.51 (8)	H10A—C10—H10B	108.1
N1—Ni1—Ni1^i^	133.62 (9)	N2—C11—C4^i^	112.7 (3)
O3—Ni1—Ni1^i^	97.29 (7)	N2—C11—H11A	109.0
O2—Ni1—Ni1^i^	131.44 (7)	C4^i^—C11—H11A	109.0
C2—C1—H1A	109.5	N2—C11—H11B	109.0
C2—C1—H1B	109.5	C4^i^—C11—H11B	109.0
H1A—C1—H1B	109.5	H11A—C11—H11B	107.8
C2—C1—H1C	109.5	C8—N1—C9	113.0 (3)
H1A—C1—H1C	109.5	C8—N1—Ni1	112.8 (2)
H1B—C1—H1C	109.5	C9—N1—Ni1	103.2 (2)
C3—C2—C6	117.3 (3)	C8—N1—HN1	108 (4)
C3—C2—C1	121.5 (4)	C9—N1—HN1	108 (4)
C6—C2—C1	121.2 (4)	Ni1—N1—HN1	112 (4)
C2—C3—C4	123.0 (3)	C11—N2—C10	115.1 (3)
C2—C3—H3	118.5	C11—N2—Ni1	106.0 (2)
C4—C3—H3	118.5	C10—N2—Ni1	108.2 (2)
C3—C4—C5	118.4 (3)	C11—N2—HN2	107 (4)
C3—C4—C11^i^	118.1 (3)	C10—N2—HN2	110 (4)
C5—C4—C11^i^	123.5 (3)	Ni1—N2—HN2	110 (4)
O1—C5—C4	122.9 (3)	O4—N3—O3	121.5 (3)
O1—C5—C7	118.0 (3)	O4—N3—O2	121.9 (3)
C4—C5—C7	119.1 (3)	O3—N3—O2	116.6 (3)
C2—C6—C7	122.2 (3)	C5—O1—Ni1	113.40 (19)
C2—C6—H6	118.9	C5—O1—Ni1^i^	125.52 (19)
C7—C6—H6	118.9	Ni1—O1—Ni1^i^	95.83 (9)
C6—C7—C5	119.4 (3)	N3—O2—Ni1	90.8 (2)
C6—C7—C8	121.7 (3)	N3—O3—Ni1	93.11 (19)
C5—C7—C8	118.7 (3)		
			
C6—C2—C3—C4	−3.1 (5)	O2—Ni1—N2—C11	32.8 (2)
C1—C2—C3—C4	176.2 (4)	Ni1^i^—Ni1—N2—C11	−101.34 (19)
C2—C3—C4—C5	−3.7 (5)	O1—Ni1—N2—C10	92.5 (2)
C2—C3—C4—C11^i^	175.9 (3)	O1^i^—Ni1—N2—C10	176.3 (2)
C3—C4—C5—O1	−172.4 (3)	N1—Ni1—N2—C10	1.1 (2)
C11^i^—C4—C5—O1	8.1 (5)	O3—Ni1—N2—C10	−95.6 (4)
C3—C4—C5—C7	7.8 (5)	O2—Ni1—N2—C10	−91.1 (2)
C11^i^—C4—C5—C7	−171.7 (3)	Ni1^i^—Ni1—N2—C10	134.7 (2)
C3—C2—C6—C7	5.8 (5)	C4—C5—O1—Ni1	−120.4 (3)
C1—C2—C6—C7	−173.5 (4)	C7—C5—O1—Ni1	59.4 (3)
C2—C6—C7—C5	−1.7 (5)	C4—C5—O1—Ni1^i^	−4.0 (4)
C2—C6—C7—C8	173.8 (3)	C7—C5—O1—Ni1^i^	175.8 (2)
O1—C5—C7—C6	174.9 (3)	O1^i^—Ni1—O1—C5	132.9 (2)
C4—C5—C7—C6	−5.2 (5)	N2—Ni1—O1—C5	−139.9 (2)
O1—C5—C7—C8	−0.7 (5)	N1—Ni1—O1—C5	−53.0 (2)
C4—C5—C7—C8	179.1 (3)	O3—Ni1—O1—C5	42.4 (2)
C6—C7—C8—N1	123.4 (4)	O2—Ni1—O1—C5	49.8 (3)
C5—C7—C8—N1	−61.0 (4)	Ni1^i^—Ni1—O1—C5	132.9 (2)
N1—C9—C10—N2	−48.9 (4)	O1^i^—Ni1—O1—Ni1^i^	0.0
C7—C8—N1—C9	166.4 (3)	N2—Ni1—O1—Ni1^i^	87.17 (11)
C7—C8—N1—Ni1	49.9 (4)	N1—Ni1—O1—Ni1^i^	174.08 (11)
C10—C9—N1—C8	−76.0 (4)	O3—Ni1—O1—Ni1^i^	−90.44 (10)
C10—C9—N1—Ni1	46.1 (3)	O2—Ni1—O1—Ni1^i^	−83.0 (3)
N2—Ni1—N1—C8	96.4 (2)	O4—N3—O2—Ni1	−179.5 (4)
O3—Ni1—N1—C8	−100.4 (2)	O3—N3—O2—Ni1	−0.4 (3)
O2—Ni1—N1—C8	−160.1 (2)	O1—Ni1—O2—N3	−8.2 (4)
Ni1^i^—Ni1—N1—C8	4.7 (3)	O1^i^—Ni1—O2—N3	−89.6 (2)
N2—Ni1—N1—C9	−25.8 (2)	N2—Ni1—O2—N3	−178.2 (2)
O3—Ni1—N1—C9	137.3 (2)	N1—Ni1—O2—N3	94.4 (2)
O2—Ni1—N1—C9	77.6 (2)	O3—Ni1—O2—N3	0.24 (19)
Ni1^i^—Ni1—N1—C9	−117.5 (2)	Ni1^i^—Ni1—O2—N3	−71.0 (2)
C4^i^—C11—N2—C10	−169.3 (3)	O4—N3—O3—Ni1	179.5 (4)
C4^i^—C11—N2—Ni1	71.2 (3)	O2—N3—O3—Ni1	0.4 (3)
C9—C10—N2—C11	−94.0 (4)	O1—Ni1—O3—N3	176.7 (2)
C9—C10—N2—Ni1	24.3 (4)	O1^i^—Ni1—O3—N3	92.3 (2)
O1—Ni1—N2—C11	−143.54 (19)	N2—Ni1—O3—N3	4.8 (5)
O1^i^—Ni1—N2—C11	−59.7 (2)	N1—Ni1—O3—N3	−90.7 (2)
N1—Ni1—N2—C11	125.1 (2)	O2—Ni1—O3—N3	−0.24 (19)
O3—Ni1—N2—C11	28.3 (5)	Ni1^i^—Ni1—O3—N3	134.08 (19)

Symmetry codes: (i) −*x*+2/3, −*y*+1/3, −*z*+1/3.
